# Comparison of Various Modalities in the Treatment of Early Knee Osteoarthritis: An Unsolved Controversy

**DOI:** 10.7759/cureus.33630

**Published:** 2023-01-11

**Authors:** Vyshnav Srinivasan, Prabhu Ethiraj, Sandesh Agarawal, Arun H S, Madhavan Parmanantham

**Affiliations:** 1 Orthopaedics, Sri Devaraj Urs Medical College, Sri Devaraj Urs Academy Of Higher Education and Research, Kolar, IND

**Keywords:** microfracture, osteoarthritis knee, hyaluronic acid, prp, knee arthroscopy

## Abstract

Introduction

Osteoarthritis (OA) of the knee is a common degenerative disease, relatively more prevalent among middle-aged people. It is one of the major reasons for walking-related disability. Recently, early knee OA has been seen as an imperative concern in many younger patients who struggle with the disabling effect of pain and management is extremely speckled. Degenerative changes such as loss of cartilage, subchondral bone changes, synovial inflammation, and meniscal degeneration are seen in OA. Symptoms are relieved by therapeutic strategies such as lifestyle behaviour changes, exercise, and oral and injectable medications. Intra-articular delivery of drugs acts as a direct effect on the target tissue, which grossly reduces side effects and is commonly preferred nowadays. The current study is a comparative assessment of the functional outcomes associated with various treatment modalities in osteoarthritis of the knee, i.e., arthroscopic debridement, arthroscopic debridement with microfracture, platelet-rich plasma (PRP) injection, and hyaluronic acid.

Methods

A retrospective observational hospital-based study was conducted among 139 cases of osteoarthritis. Patients aged between 40-60 years with diagnosed Kellgren- Lawrence grade 1 and 2 OA knee, who underwent arthroscopic debridement, arthroscopic debridement with microfracture, PRP injection, or hyaluronic acid in our institute were included.

Results

The mean age was 52.83 + 6.8 years. The mean BMI was 27.45 + 1.6 kg/m^2^. At the time of diagnosis of OA, the mean visual analogue scale for pain (VAS) and Western Ontario and McMaster Universities Arthritis Index (WOMAC) scores were 7.26 +0.7 and 55.30 + 2.21 respectively. Out of the total, 88 (63.3%) were females and 51 (36.7%) were males. Right-sided OA knee was seen in the majority of study participants. Of the total, 93 (66.9%) patients had grade 2 and only 46 (33.1%) had grade 1 OA. A statistically significant difference was found between the mean VAS and WOMAC score at the time of diagnosis, three weeks, three months, as well as at six months of therapy. In the hyaluronic acid treatment, no significant difference was found in mean VAS and WOMAC scores.

Conclusion

Various treatments are available for early-diagnosed OA. According to the findings of this study, overall improvement was seen in VAS and WOMAC scores at the follow-up after six months of specific treatment. In a period over six months, arthroscopic debridement with micro-fracture was more effective and safe when compared with other modalities of treatment for early OA knee. Also, injection of PRP was superior to other methods for VAS pain reduction, and WOMAC-pain and WOMAC-stiffness scores improved at one month.

## Introduction

Osteoarthritis (OA) of the knee is a common degenerative disease, relatively more prevalent among middle-aged people. It is one of the major reasons for walking-related disability. Recently, early knee OA (Kellgren-Lawrence (KL) grade 1 and 2) is becoming an imperative concern in many younger patients who struggle with the disabling effect of pain, and management is extremely speckled. Degenerative changes such as loss of cartilage, subchondral bone changes, synovial inflammation, and meniscal degeneration are seen in OA. These changes are early identified with the help of MRI and ultrasound as well as extensive use of arthroscopy in which there is direct visualization of the whole area of the affected joint and also of the cartilage, menisci, synovial membrane, ligaments, and fat pad [1].

Globally, it is the leading cause of chronic disability in patients older than 70 years and has been deputed as a ‘prior disease’ by the WHO. OA is considered one of the most disabling diseases in developing countries like India [2]. According to WHO, it is estimated that globally, 18.0% of women aged over 60 and 9.6% of men over 60 years suffer from OA. Eighty percent of people who suffer from OA have a restricted range of motion and 25% have difficulty in their day-to-day activities [3]. The prevalence of this condition is increasing day by day due to the demographic burden of the population in developed and developing countries along with an increase in various age-related risk factors [4,5].

Even though symptomatic knee OA differs greatly in affected individuals, ruling out early knee OA is critical for evading or delaying the progression to a severe stage of the disease. When patients present with early OA at a younger age, it becomes a serious challenge for orthopaedics consultants, because of a combination of high functional and motility demands and restricted management options [6].

In the management of OA, symptoms are relieved by therapeutic strategies such as lifestyle behaviour changes, exercise, and oral and injectable medications [7]. Oral medications such as non-steroidal anti-inflammatory drugs (NSAIDs) and paracetamol are effective in relieving pain in this condition [8]. Because of the long-term adverse effects of these oral medications on the gastrointestinal, cardiovascular, and renal systems, the use of oral medication has been reduced comparatively [9].

Intra-articular injectable drugs are most commonly used nowadays because of their direct effect on the target tissues and reduced adverse effects as it's delivered directly. The majority of injectable medications are corticosteroids (CS), hyaluronic acid (HA), polynucleotides, oxygen-ozone therapy, platelet-rich plasma (PRP), and mesenchymal stem cells (MSCs). CS and HA are mentioned as the best treatments in a few studies for early OA knee [10].

Newer drugs like PRP and MSCs have limited literature regarding their safety and efficacy. These medications play a beneficial role in reducing local inflammation and promoting cartilage and synovial anabolism by releasing a series of growth factors and immune-modulatory molecules [11]. PRP, which is the concentrated platelets in a small volume of blood plasma, is widely used in clinical and tissue engineering research. The mechanism of action of PRP is by increasing the concentration of growth factors such as platelet-derived growth factor (PDGF), basic fibroblast growth factor (bFGF), transforming growth factor ß (TGF-ß), vascular endothelial growth factor (VEGF), interleukins, hormones, and several hundred other proteins that are released by platelets, because of which healing is accelerated [12]. Arthroscopic debridement with microfracture is a technically easy, safe, and cost-effective treatment option for articular cartilage lesions of the knee [13]. So, the current study was conducted to assess and compare the functional outcomes associated with various treatment modalities in osteoarthritis of the knee, i.e., arthroscopic debridement, arthroscopic debridement with microfracture, PRP injection, and HA.

## Materials and methods

This retrospective study included 139 patients who were diagnosed with OA knee and underwent surgical treatment such as arthroscopic debridement (n=38) and arthroscopic debridement with microfracture (n=32) and conservative management such as PEP injection (n=39) and HA (n=30), who visited the Department of Orthopaedics in RL Jalappa Hospital, Kolar, Karnataka, India, from August 2018 to August 2022. Data collection was performed after obtaining ethical approval from the Institutional Ethics Committee of Sri Devaraj Urs Medical College, Kolar, Karnataka, India (approval number: DMC/KLR/IEC/197/2022-23). Patients aged 40-60 years with diagnosed KL grade 1 and 2 OA knee who underwent arthroscopic debridement, arthroscopic debridement with microfracture, PRP injection, or HA in our institute were included. The exclusion criteria were patients with previous lower extremity surgery, previous knee trauma, poor diabetic control with glycated haemoglobin (HBA1c) of more than 8%, rheumatoid arthritis, bilateral OA knee, gout, and peripheral neuropathy.

Patient data were collected from the clinical follow-up notes. The following data were taken from clinical notes: general physical examination, knee joint examination (inspection, palpation, range of movements, measurements, and special tests), grade of OA of the knee (weight-bearing anteroposterior view and lateral views), routine investigation, treatment modalities (arthroscopic debridement/arthroscopic debridement with microfracture/PRP injection/HA), complications, and outcomes based on a scoring system (visual analogue scale for pain (VAS) and Western Ontario and McMaster Universities Arthritis Index (WOMAC)).

Data were entered into an Microsoft Excel sheet (Microsoft Corporation, Redmond, Washington, United States) and analyzed using IBM SPSS Statistics for Windows, Version 24.0 (Released 2016; IBM Corp., Armonk, New York, United States). Continuous variables were summarized using mean with SD. Categorical variables such as sex and KL grading were summarised using frequency and percentages. Functional outcomes measured in terms of VAS and WOMAC scores for pain, stiffness, and physical function were described using mean and SD. A comparison of outcomes between the four study groups (arthroscopic debridement, arthroscopic debridement with microfracture, PRP injection, and HA) was done by using one-way ANOVA test. p-value less than 0.05 was considered statistically significant

## Results

A total of 139 patients were included in the study, of which 38 underwent arthroscopic debridement, 32 underwent arthroscopic debridement with microfracture, 39 were treated with PEP injection, and 30 were treated with HA. Of the 139 patients, only 69 had a history of comorbidity; 39 (56.5%) had a history of diabetes and 30 (43.4%) had a history of hypertension (Table 1).

**Table 1:** Comorbidities distribution seen among study participants (n=69)

Comorbidities	Frequency (%)
Diabetes	39 (56.5%)
Hypertension	30 (43.4%)

A statistically significant difference was found between the mean VAS and WOMAC scores in all the groups except in the HA group at the time of diagnosis, three weeks, three months, as well as at six months of therapy. In the HA treatment group, no significant difference was found in the mean VAS and WOMAC scores (Table 2).

**Table 2:** Mean VAS and WOMAC scores among study participants VAS: visual analogue scale for pain; WOMAC: Western Ontario and McMaster Universities Arthritis Index

Type of Treatment		VAS score	P-value	WOMAC score	P-value
Platelet Rich Plasma (n=39)	At time of admission	6.8 + 0.72	0.002	55.43 + 2.29	0.004
	At 6 weeks	5.41 + 3.81		45.25 + 2.78	
	At 3 months	3.87 + 0.83		35.15 + 2.45	
	At 6 months	2.76 + 1.51		26.71 + 8.34	
Hyaluronic Acid (n=30)	At time of admission	7.73 + 0.44	0.214	54.93 + 2.61	0.137
	At 6 weeks	6.06 + 0.25		45.46 + 2.22	
	At 3 months	4.5 + 0.61		35.76 + 2.34	
	At 6 months	2.3 + 1.31		25.13 + 2.44	
Arthroscopic Debridement (n=38)	At time of admission	7.71 + 0.45	0.09	54.89 + 2.53	0.012
	At 6 weeks	6.05 + 0.22		45.52 + 2.25	
	At 3 months	4.55 + 0.6		35.92 + 2.28	
	At 6 months	2.18 + 1.27		24.94 + 2.57	
Arthroscopic Debridement + Microfracture (n=32)	At time of admission	6.84 + 0.51	0.0001	56.01 + 2.1	0.003
	At 6 weeks	5.68 + 0.85		46.18 + 2.23	
	At 3 months	4.25 + 0.80		36.5 + 2.09	
	At 6 months	2.78 + 1.31		23.02 + 4.06	

The mean age was 52.83 + 6.8 years. The mean BMI was 27.45 + 1.6 kg/m2. At the time of diagnosis of OA, mean VAS and WOMAC scores were 7.26 +0.7 and 55.30 + 2.21, respectively (Table 3).

**Table 3:** Distribution of various details of study participants (n=139) VAS: visual analogue scale for pain; WOMAC: Western Ontario and McMaster Universities Arthritis Index

Variable	Mean + SD
Age	52.83 + 6.8
BMI	27.45 + 1.6
VAS score at the time of diagnosis	7.26 +0.7
WOMAC score the time of diagnosis	55.30 + 2.21

## Discussion

In the present study, the mean age was 52.83 + 6.8 years. Similar age group patients were involved in the study conducted by Li et al. [14] and Kon et al. [15]. The mean BMI was 27.45 + 1.6 kg/m^2^. At the time of diagnosis of OA, mean VAS and WOMAC scores were 7.26 +0.7 and 55.30 + 2.21, respectively. Out of the total, 88 (63.3%) were females and 51 (36.7%) were males in the study, while in a study conducted by Scher et al. [16] and Sihvonen et al. [17], more female participants were affected than males.

In our study, the majority of study participants were affected on the right side of the knee joint. In total, 93 (66.9%) patients had grade 2 and only 46 (33.1%) had grade 1 OA. Similar findings were seen in studies done by Di Matteo et al. [18] and Wang et al. [19]. However, in a study by Evans et al. [20], most participants were affected on the left side of the knee joint.

Statistically significant difference was found in this study between the mean VAS and mean WOMAC scores at the time of diagnosis, three weeks, three months, as well as at six months, except in HA treatment, where no significant difference was found between mean VAS and WOMAC scores. Similar findings were seen in the studies by Beaufils et al. [21], Guenoun et al. [22], and Karasavvidis et al. [23].

In a study conducted by Sun et al., mean VAS scores were improved by 21.9 mm, 25.8 mm, and 20.4 mm from baseline at the one, three, and six-month follow-ups in the only on-type injection group, whereas the VAS scores improved by 18.1 mm, 28.1 mm, and 31.8 mm from baseline in the combined-injection group [24]. The between-group comparison showed that patients receiving one injection of PRP experienced significantly greater VAS pain reduction than patients receiving arthroscopic debridement with microfracture at the one-month follow-up. However, at the six-month follow-up, the arthroscopic debridement with microfracture group achieved significantly better VAS pain reduction than the other groups.

The study of Szwedowski et al. stated that the mechanism of actions in the joint to optimize and standardize PRP formulations, identify the most suitable biomarkers for assessing treatment efficacy, and reveal the underlying mechanisms are the major challenges in knee OA treatment with PRP intra-articular injections that are involved in OA pathophysiology [25]. Also, different PRP formulations are available on the market; so, dosage level and efficacy can be checked before being given as a treatment.

Everts et al. conducted a study in which the full potential of PRP technology, the concept of individualized treatment protocols based on bioformulation options, and platelet dosing, angiogenesis, and antimicrobial and painkilling effects of PRP relevant to orthopaedic surgery have been addressed [26]. In the study of Hahn et al., cell number and metabolic cell activity gradually increase the dose- and time-dependence of human chondrocytes within 14 days [27]. Furthermore, cells revealed a significant increase in the amount of bone-specific procollagen type 1 and sulfated glycosaminoglycans (14 days, 2.69-fold); however, no significant change was observed in the amount of cartilage-specific collagen type 2.

In a study by Bowman et al., HA injection provided good pain relief and functional outcomes and was considered the best conservative line for hip OA [28]. A randomized clinical trial by Battaglia et al. compared HA injection to PRP injection and stated that PRP is inferior to HA in the relief of pain and symptoms in patients with OA knee [29]. In 2003, Vad et al. reported that rapid pain relief for hip OA in mild to moderate cases is seen with intra-articular HA injection [30]. Apart from intra-articular injection of low and high molecular weight HA being notably effective in relieving pain, it was also associated with a reduction of 48.2% of NSAID consumption in the third month when compared with baseline values [31].

In the study by Katz et al., they state that the popularity of arthroscopic knee surgery rose quickly because of advanced technologies [32]. Also, there is an increase in the number of procedures performed, especially arthroscopic debridement for OA and arthroscopic partial meniscectomy for degenerative meniscal tear, which outstripped the rigorous tracking of procedure outcomes. Mancò et al. found that PRP combined with microfracture yielded better clinical and functional results than only arthroscopy debridement with microfracture at the short-term follow-up with regard to pain, but at follow-up after two years, the clinical results were similar [33]. Manunta and Manconi found that the difference between the VAS scores of patients treated with microfracture plus PRP and microfracture alone was not significant [34].

The current study has certain limitations. The study is retrospective, with a low sample size and shorter follow-up, and only the records that were available were used. Another limitation of this study was that the influence of systemic diseases such as diabetes and hypertension on degenerative conditions of joints was not accounted for.

## Conclusions

Various treatments are available for early diagnosed OA. In this study, overall improvement was seen in VAS and WOMAC scores at the follow-up after six months of specific treatment. In the period over six months, when compared with other modalities of treatment for early OA knee, arthroscopic debridement with microfracture was found to be more effective and safe. Also, injection of PRP was superior to other methods for VAS pain reduction, and WOMAC-pain and WOMAC-stiffness scores improved at one month.
